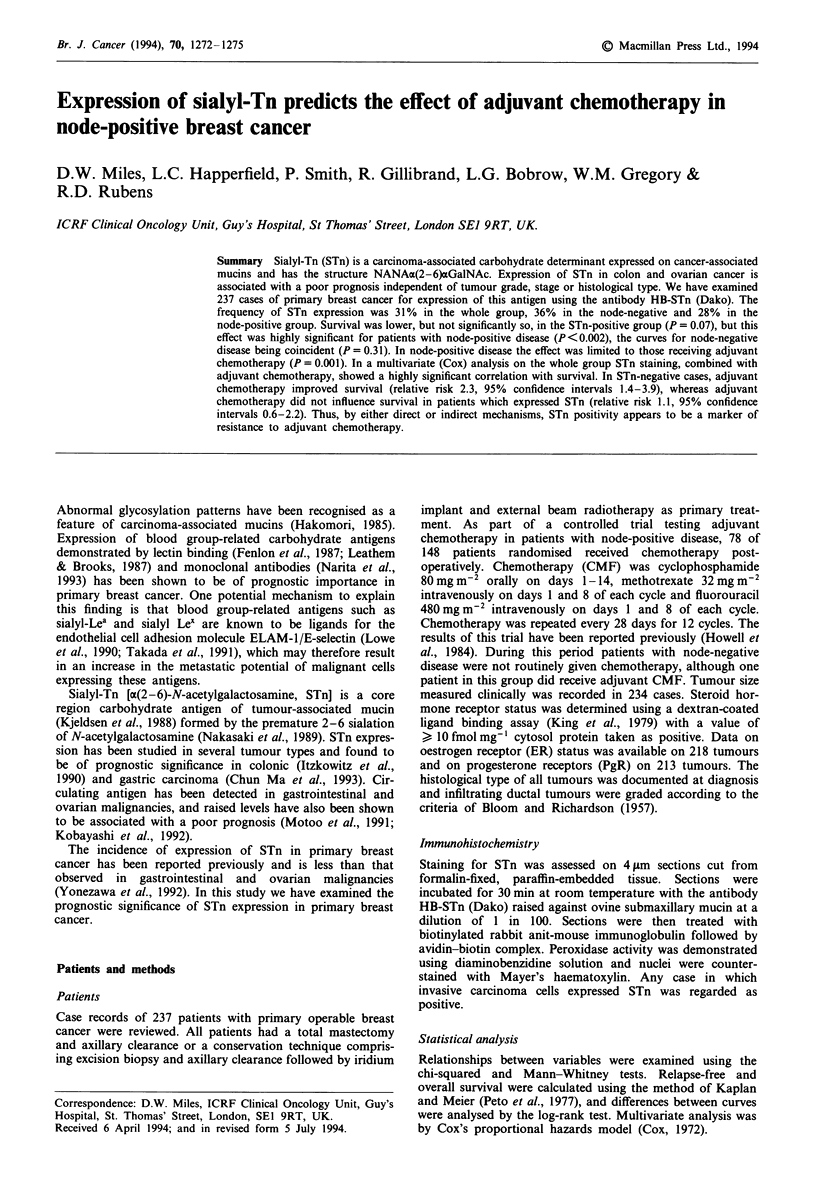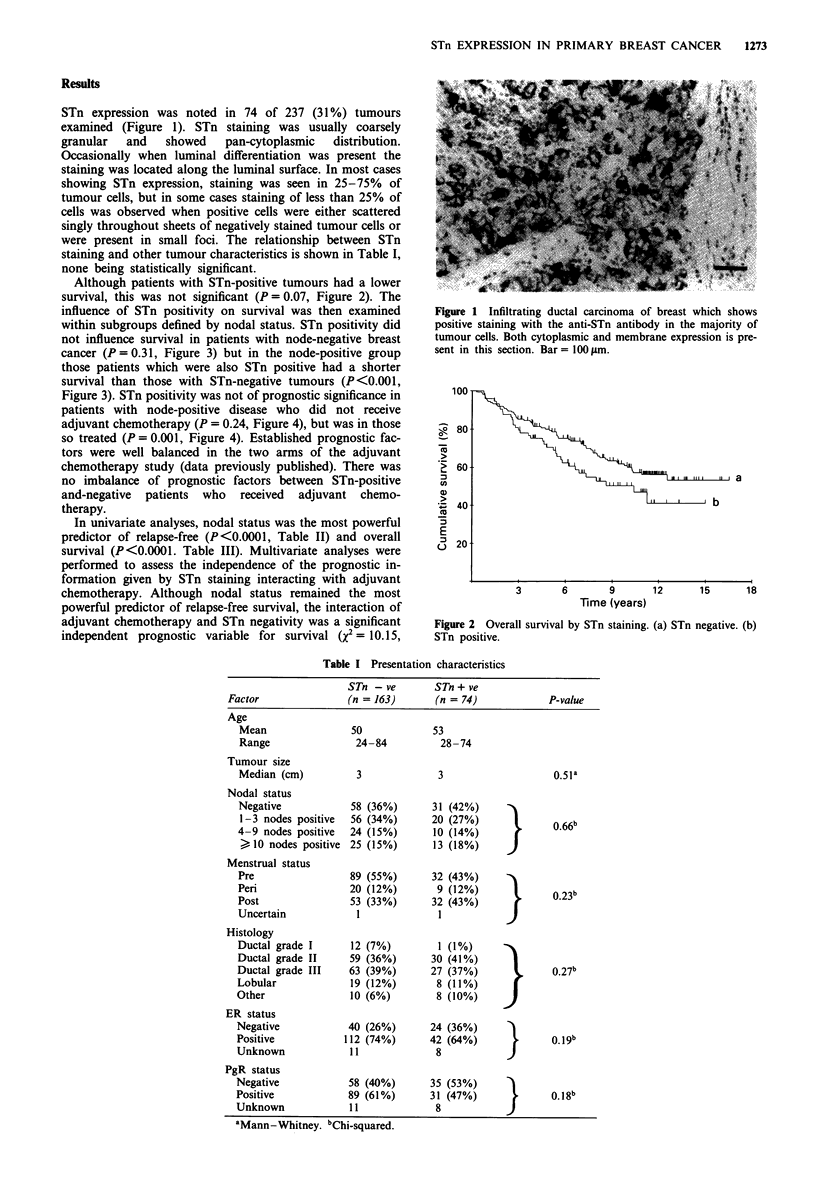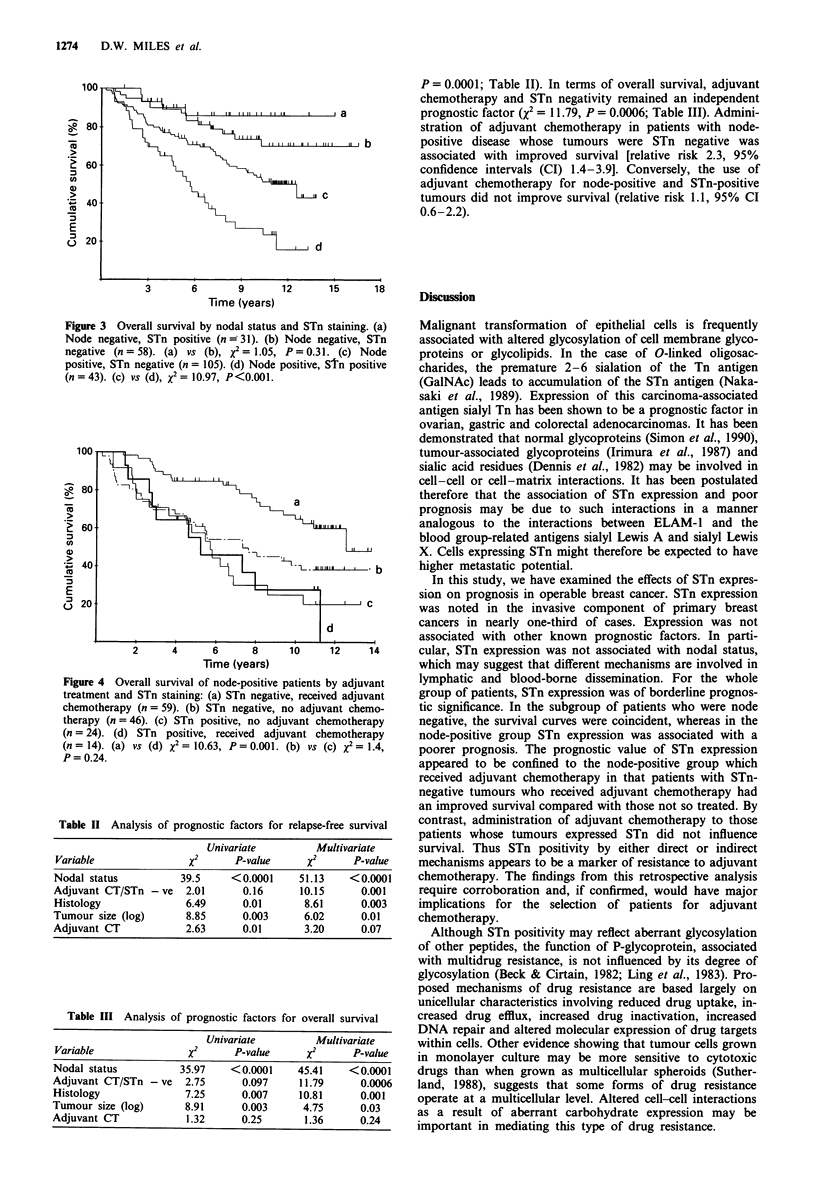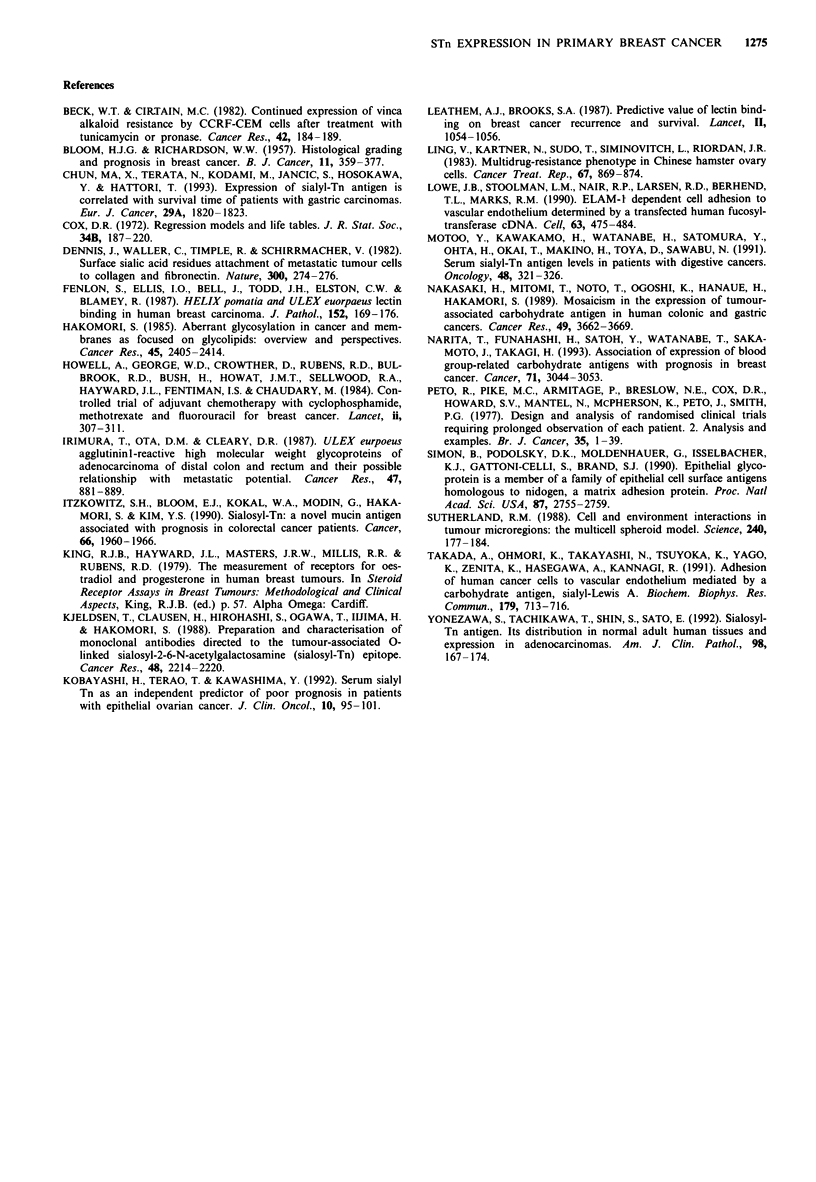# Expression of sialyl-Tn predicts the effect of adjuvant chemotherapy in node-positive breast cancer.

**DOI:** 10.1038/bjc.1994.486

**Published:** 1994-12

**Authors:** D. W. Miles, L. C. Happerfield, P. Smith, R. Gillibrand, L. G. Bobrow, W. M. Gregory, R. D. Rubens

**Affiliations:** ICRF Clinical Oncology Unit, Guy's Hospital, London, UK.

## Abstract

**Images:**


Br. J. Cancer (1994), 70, 1272–1275 © Macmillan Press Ltd., 1994

### Expression of sialyl-Tn predicts the effect of adjuvant chemotherapy in node-positive breast cancer

D.W. Miles, L.C. Happerfield, P. Smith, R. Gillibrand, L.G. Bobrow, W.M. Gregory & R.D. Rubens

*ICRF Clinical Oncology Unit, Guy's Hospital, St Thomas' Street, London SE1 9RT, UK.*

**Summary** Sialyl-Tn (STn) is a carcinoma-associated carbohydrate determinant expressed on cancer-associated mucins and has the structure NANAα(2–6)αGalNAc. Expression of STn in colon and ovarian cancer is associated with a poor prognosis independent of tumour grade, stage or histological type. We have examined 237 cases of primary breast cancer for expression of this antigen using the antibody HB-STn (Dako). The frequency of STn expression was 31% in the whole group, 36% in the node-negative and 28% in the node-positive group. Survival was lower, but not significantly so, in the STn-positive group ($P = 0.07$), but this effect was highly significant for patients with node-positive disease ($P < 0.002$), the curves for node-negative disease being coincident ($P = 0.31$). In node-positive disease the effect was limited to those receiving adjuvant chemotherapy ($P = 0.001$). In a multivariate (Cox) analysis on the whole group STn staining, combined with adjuvant chemotherapy, showed a highly significant correlation with survival. In STn-negative cases, adjuvant chemotherapy improved survival (relative risk 2.3, 95% confidence intervals 1.4–3.9), whereas adjuvant chemotherapy did not influence survival in patients which expressed STn (relative risk 1.1, 95% confidence intervals 0.6–2.2). Thus, by either direct or indirect mechanisms, STn positivity appears to be a marker of resistance to adjuvant chemotherapy.

Abnormal glycosylation patterns have been recognised as a feature of carcinoma-associated mucins (Hakomori, 1985). Expression of blood group-related carbohydrate antigens demonstrated by lectin binding (Fenlon *et al.*, 1987; Leathem & Brooks, 1987) and monoclonal antibodies (Narita *et al.*, 1993) has been shown to be of prognostic importance in primary breast cancer. One potential mechanism to explain this finding is that blood group-related antigens such as sialyl-$Le^a$ and sialyl $Le^x$ are known to be ligands for the endothelial cell adhesion molecule ELAM-1/E-selectin (Lowe *et al.*, 1990; Takada *et al.*, 1991), which may therefore result in an increase in the metastatic potential of malignant cells expressing these antigens.

Sialyl-Tn [α(2–6)-*N*-acetylgalactosamine, STn] is a core region carbohydrate antigen of tumour-associated mucin (Kjeldsen *et al.*, 1988) formed by the premature 2–6 sialation of *N*-acetylgalactosamine (Nakasaki *et al.*, 1989). STn expression has been studied in several tumour types and found to be of prognostic significance in colonic (Itzkowitz *et al.*, 1990) and gastric carcinoma (Chun Ma *et al.*, 1993). Circulating antigen has been detected in gastrointestinal and ovarian malignancies, and raised levels have also been shown to be associated with a poor prognosis (Motoo *et al.*, 1991; Kobayashi *et al.*, 1992).

The incidence of expression of STn in primary breast cancer has been reported previously and is less than that observed in gastrointestinal and ovarian malignancies (Yonezawa *et al.*, 1992). In this study we have examined the prognostic significance of STn expression in primary breast cancer.

### Patients and methods

### *Patients*

Case records of 237 patients with primary operable breast cancer were reviewed. All patients had a total mastectomy and axillary clearance or a conservation technique comprising excision biopsy and axillary clearance followed by iridium implant and external beam radiotherapy as primary treatment. As part of a controlled trial testing adjuvant chemotherapy in patients with node-positive disease, 78 of 148 patients randomised received chemotherapy post-operatively. Chemotherapy (CMF) was cyclophosphamide $80\,mg\,m^{-2}$ orally on days 1–14, methotrexate $32\,mg\,m^{-2}$ intravenously on days 1 and 8 of each cycle and fluorouracil $480\,mg\,m^{-2}$ intravenously on days 1 and 8 of each cycle. Chemotherapy was repeated every 28 days for 12 cycles. The results of this trial have been reported previously (Howell *et al.*, 1984). During this period patients with node-negative disease were not routinely given chemotherapy, although one patient in this group did receive adjuvant CMF. Tumour size measured clinically was recorded in 234 cases. Steroid hormone receptor status was determined using a dextran-coated ligand binding assay (King *et al.*, 1979) with a value of $\geqslant 10\,fmol\,mg^{-1}$ cytosol protein taken as positive. Data on oestrogen receptor (ER) status was available on 218 tumours and on progesterone receptors (PgR) on 213 tumours. The histological type of all tumours was documented at diagnosis and infiltrating ductal tumours were graded according to the criteria of Bloom and Richardson (1957).

### *Immunohistochemistry*

Staining for STn was assessed on 4 μm sections cut from formalin-fixed, paraffin-embedded tissue. Sections were incubated for 30 min at room temperature with the antibody HB-STn (Dako) raised against ovine submaxillary mucin at a dilution of 1 in 100. Sections were then treated with biotinylated rabbit anit-mouse immunoglobulin followed by avidin–biotin complex. Peroxidase activity was demonstrated using diaminobenzidine solution and nuclei were counterstained with Mayer's haematoxylin. Any case in which invasive carcinoma cells expressed STn was regarded as positive.

### *Statistical analysis*

Relationships between variables were examined using the chi-squared and Mann–Whitney tests. Relapse-free and overall survival were calculated using the method of Kaplan and Meier (Peto *et al.*, 1977), and differences between curves were analysed by the log-rank test. Multivariate analysis was by Cox's proportional hazards model (Cox, 1972).

Correspondence: D.W. Miles, ICRF Clinical Oncology Unit, Guy's Hospital, St. Thomas' Street, London, SE1 9RT, UK.
Received 6 April 1994; and in revised form 5 July 1994.

### Results

STn expression was noted in 74 of 237 (31%) tumours examined (Figure 1). STn staining was usually coarsely granular and showed pan-cytoplasmic distribution. Occasionally when luminal differentiation was present the staining was located along the luminal surface. In most cases showing STn expression, staining was seen in 25–75% of tumour cells, but in some cases staining of less than 25% of cells was observed when positive cells were either scattered singly throughout sheets of negatively stained tumour cells or were present in small foci. The relationship between STn staining and other tumour characteristics is shown in Table I, none being statistically significant.

Although patients with STn-positive tumours had a lower survival, this was not significant ($P = 0.07$, Figure 2). The influence of STn positivity on survival was then examined within subgroups defined by nodal status. STn positivity did not influence survival in patients with node-negative breast cancer ($P = 0.31$, Figure 3) but in the node-positive group those patients which were also STn positive had a shorter survival than those with STn-negative tumours ($P < 0.001$, Figure 3). STn positivity was not of prognostic significance in patients with node-positive disease who did not receive adjuvant chemotherapy ($P = 0.24$, Figure 4), but was in those so treated ($P = 0.001$, Figure 4). Established prognostic factors were well balanced in the two arms of the adjuvant chemotherapy study (data previously published). There was no imbalance of prognostic factors between STn-positive and-negative patients who received adjuvant chemotherapy.

In univariate analyses, nodal status was the most powerful predictor of relapse-free ($P < 0.0001$, Table II) and overall survival ($P < 0.0001$. Table III). Multivariate analyses were performed to assess the independence of the prognostic information given by STn staining interacting with adjuvant chemotherapy. Although nodal status remained the most powerful predictor of relapse-free survival, the interaction of adjuvant chemotherapy and STn negativity was a significant independent prognostic variable for survival ($\chi^2 = 10.15$,

**Figure 1** Infiltrating ductal carcinoma of breast which shows positive staining with the anti-STn antibody in the majority of tumour cells. Both cytoplasmic and membrane expression is present in this section. Bar = 100 μm.

**Figure 2** Overall survival by STn staining. (a) STn negative. (b) STn positive.

**Table I** Presentation characteristics

| Factor | STn − ve (n = 163) | STn + ve (n = 74) | P-value |
|---|---|---|---|
| Age | | | |
| Mean | 50 | 53 | |
| Range | 24–84 | 28–74 | |
| Tumour size | | | |
| Median (cm) | 3 | 3 | 0.51[a] |
| Nodal status | | | 0.66[b] |
| Negative | 58 (36%) | 31 (42%) | |
| 1–3 nodes positive | 56 (34%) | 20 (27%) | |
| 4–9 nodes positive | 24 (15%) | 10 (14%) | |
| ⩾10 nodes positive | 25 (15%) | 13 (18%) | |
| Menstrual status | | | 0.23[b] |
| Pre | 89 (55%) | 32 (43%) | |
| Peri | 20 (12%) | 9 (12%) | |
| Post | 53 (33%) | 32 (43%) | |
| Uncertain | 1 | 1 | |
| Histology | | | 0.27[b] |
| Ductal grade I | 12 (7%) | 1 (1%) | |
| Ductal grade II | 59 (36%) | 30 (41%) | |
| Ductal grade III | 63 (39%) | 27 (37%) | |
| Lobular | 19 (12%) | 8 (11%) | |
| Other | 10 (6%) | 8 (10%) | |
| ER status | | | 0.19[b] |
| Negative | 40 (26%) | 24 (36%) | |
| Positive | 112 (74%) | 42 (64%) | |
| Unknown | 11 | 8 | |
| PgR status | | | 0.18[b] |
| Negative | 58 (40%) | 35 (53%) | |
| Positive | 89 (61%) | 31 (47%) | |
| Unknown | 11 | 8 | |

[a]Mann–Whitney. [b]Chi-squared.

**Figure 3** Overall survival by nodal status and STn staining. (a) Node negative, STn positive ($n = 31$). (b) Node negative, STn negative ($n = 58$). (a) *vs* (b), $\chi^2 = 1.05$, $P = 0.31$. (c) Node positive, STn negative ($n = 105$). (d) Node positive, STn positive ($n = 43$). (c) *vs* (d), $\chi^2 = 10.97$, $P < 0.001$.

**Figure 4** Overall survival of node-positive patients by adjuvant treatment and STn staining: (a) STn negative, received adjuvant chemotherapy ($n = 59$). (b) STn negative, no adjuvant chemotherapy ($n = 46$). (c) STn positive, no adjuvant chemotherapy ($n = 24$). (d) STn positive, received adjuvant chemotherapy ($n = 14$). (a) *vs* (d) $\chi^2 = 10.63$, $P = 0.001$. (b) *vs* (c) $\chi^2 = 1.4$, $P = 0.24$.

**Table II** Analysis of prognostic factors for relapse-free survival

| Variable | Univariate $\chi^2$ | Univariate P-value | Multivariate $\chi^2$ | Multivariate P-value |
|---|---|---|---|---|
| Nodal status | 39.5 | <0.0001 | 51.13 | <0.0001 |
| Adjuvant CT/STn – ve | 2.01 | 0.16 | 10.15 | 0.001 |
| Histology | 6.49 | 0.01 | 8.61 | 0.003 |
| Tumour size (log) | 8.85 | 0.003 | 6.02 | 0.01 |
| Adjuvant CT | 2.63 | 0.01 | 3.20 | 0.07 |

**Table III** Analysis of prognostic factors for overall survival

| Variable | Univariate $\chi^2$ | Univariate P-value | Multivariate $\chi^2$ | Multivariate P-value |
|---|---|---|---|---|
| Nodal status | 35.97 | <0.0001 | 45.41 | <0.0001 |
| Adjuvant CT/STn – ve | 2.75 | 0.097 | 11.79 | 0.0006 |
| Histology | 7.25 | 0.007 | 10.81 | 0.001 |
| Tumour size (log) | 8.91 | 0.003 | 4.75 | 0.03 |
| Adjuvant CT | 1.32 | 0.25 | 1.36 | 0.24 |

$P = 0.0001$; Table II). In terms of overall survival, adjuvant chemotherapy and STn negativity remained an independent prognostic factor ($\chi^2 = 11.79$, $P = 0.0006$; Table III). Administration of adjuvant chemotherapy in patients with node-positive disease whose tumours were STn negative was associated with improved survival [relative risk 2.3, 95% confidence intervals (CI) 1.4–3.9]. Conversely, the use of adjuvant chemotherapy for node-positive and STn-positive tumours did not improve survival (relative risk 1.1, 95% CI 0.6–2.2).

### Discussion

Malignant transformation of epithelial cells is frequently associated with altered glycosylation of cell membrane glycoproteins or glycolipids. In the case of *O*-linked oligosaccharides, the premature 2–6 sialation of the Tn antigen (GalNAc) leads to accumulation of the STn antigen (Nakasaki *et al.*, 1989). Expression of this carcinoma-associated antigen sialyl Tn has been shown to be a prognostic factor in ovarian, gastric and colorectal adenocarcinomas. It has been demonstrated that normal glycoproteins (Simon *et al.*, 1990), tumour-associated glycoproteins (Irimura *et al.*, 1987) and sialic acid residues (Dennis *et al.*, 1982) may be involved in cell–cell or cell–matrix interactions. It has been postulated therefore that the association of STn expression and poor prognosis may be due to such interactions in a manner analogous to the interactions between ELAM-1 and the blood group-related antigens sialyl Lewis A and sialyl Lewis X. Cells expressing STn might therefore be expected to have higher metastatic potential.

In this study, we have examined the effects of STn expression on prognosis in operable breast cancer. STn expression was noted in the invasive component of primary breast cancers in nearly one-third of cases. Expression was not associated with other known prognostic factors. In particular, STn expression was not associated with nodal status, which may suggest that different mechanisms are involved in lymphatic and blood-borne dissemination. For the whole group of patients, STn expression was of borderline prognostic significance. In the subgroup of patients who were node negative, the survival curves were coincident, whereas in the node-positive group STn expression was associated with a poorer prognosis. The prognostic value of STn expression appeared to be confined to the node-positive group which received adjuvant chemotherapy in that patients with STn-negative tumours who received adjuvant chemotherapy had an improved survival compared with those not so treated. By contrast, administration of adjuvant chemotherapy to those patients whose tumours expressed STn did not influence survival. Thus STn positivity by either direct or indirect mechanisms appears to be a marker of resistance to adjuvant chemotherapy. The findings from this retrospective analysis require corroboration and, if confirmed, would have major implications for the selection of patients for adjuvant chemotherapy.

Although STn positivity may reflect aberrant glycosylation of other peptides, the function of P-glycoprotein, associated with multidrug resistance, is not influenced by its degree of glycosylation (Beck & Cirtain, 1982; Ling *et al.*, 1983). Proposed mechanisms of drug resistance are based largely on unicellular characteristics involving reduced drug uptake, increased drug efflux, increased drug inactivation, increased DNA repair and altered molecular expression of drug targets within cells. Other evidence showing that tumour cells grown in monolayer culture may be more sensitive to cytotoxic drugs than when grown as multicellular spheroids (Sutherland, 1988), suggests that some forms of drug resistance operate at a multicellular level. Altered cell–cell interactions as a result of aberrant carbohydrate expression may be important in mediating this type of drug resistance.

### References

BECK, W.T. & CIRTAIN, M.C. (1982). Continued expression of vinca alkaloid resistance by CCRF-CEM cells after treatment with tunicamycin or pronase. *Cancer Res.*, **42**, 184-189.

BLOOM, H.J.G. & RICHARDSON, W.W. (1957). Histological grading and prognosis in breast cancer. *B. J. Cancer*, **11**, 359-377.

CHUN, MA, X., TERATA, N., KODAMI, M., JANCIC, S., HOSOKAWA, Y. & HATTORI, T. (1993). Expression of sialyl-Tn antigen is correlated with survival time of patients with gastric carcinomas. *Eur. J. Cancer*, **29A**, 1820-1823.

COX, D.R. (1972). Regression models and life tables. *J. R. Stat. Soc.*, **34B**, 187-220.

DENNIS, J., WALLER, C., TIMPLE, R. & SCHIRRMACHER, V. (1982). Surface sialic acid residues attachment of metastatic tumour cells to collagen and fibronectin. *Nature*, **300**, 274-276.

FENLON, S., ELLIS, I.O., BELL, J., TODD, J.H., ELSTON, C.W. & BLAMEY, R. (1987). *HELIX pomatia and ULEX euorpaeus* lectin binding in human breast carcinoma. *J. Pathol.*, **152**, 169-176.

HAKOMORI, S. (1985). Aberrant glycosylation in cancer and membranes as focused on glycolipids: overview and perspectives. *Cancer Res.*, **45**, 2405-2414.

HOWELL, A., GEORGE, W.D., CROWTHER, D., RUBENS, R.D., BULBROOK, R.D., BUSH, H., HOWAT, J.M.T., SELLWOOD, R.A., HAYWARD, J.L., FENTIMAN, I.S. & CHAUDARY, M. (1984). Controlled trial of adjuvant chemotherapy with cyclophosphamide, methotrexate and fluorouracil for breast cancer. *Lancet*, **ii**, 307-311.

IRIMURA, T., OTA, D.M. & CLEARY, D.R. (1987). *ULEX eurpoeus* agglutininl-reactive high molecular weight glycoproteins of adenocarcinoma of distal colon and rectum and their possible relationship with metastatic potential. *Cancer Res.*, **47**, 881-889.

ITZKOWITZ, S.H., BLOOM, E.J., KOKAL, W.A., MODIN, G., HAKAMORI, S. & KIM, Y.S. (1990). Sialosyl-Tn: a novel mucin antigen associated with prognosis in colorectal cancer patients. *Cancer*, **66**, 1960-1966.

KING, R.J.B., HAYWARD, J.L., MASTERS, J.R.W., MILLIS, R.R. & RUBENS, R.D. (1979). The measurement of receptors for oestradiol and progesterone in human breast tumours. In *Steroid Receptor Assays in Breast Tumours: Methodological and Clinical Aspects*, King, R.J.B. (ed.) p. 57. Alpha Omega: Cardiff.

KJELDSEN, T., CLAUSEN, H., HIROHASHI, S., OGAWA, T., IIJIMA, H. & HAKOMORI, S. (1988). Preparation and characterisation of monoclonal antibodies directed to the tumour-associated O-linked sialosyl-2-6-N-acetylgalactosamine (sialosyl-Tn) epitope. *Cancer Res.*, **48**, 2214-2220.

KOBAYASHI, H., TERAO, T. & KAWASHIMA, Y. (1992). Serum sialyl Tn as an independent predictor of poor prognosis in patients with epithelial ovarian cancer. *J. Clin. Oncol.*, **10**, 95-101.

LEATHEM, A.J., BROOKS, S.A. (1987). Predictive value of lectin binding on breast cancer recurrence and survival. *Lancet*, **II**, 1054-1056.

LING, V., KARTNER, N., SUDO, T., SIMINOVITCH, L., RIORDAN, J.R. (1983). Multidrug-resistance phenotype in Chinese hamster ovary cells. *Cancer Treat. Rep.*, **67**, 869-874.

LOWE, J.B., STOOLMAN, L.M., NAIR, R.P., LARSEN, R.D., BERHEND, T.L., MARKS, R.M. (1990). ELAM-1 dependent cell adhesion to vascular endothelium determined by a transfected human fucosyltransferase cDNA. *Cell*, **63**, 475-484.

MOTOO, Y., KAWAKAMO, H., WATANABE, H., SATOMURA, Y., OHTA, H., OKAI, T., MAKINO, H., TOYA, D., SAWABU, N. (1991). Serum sialyl-Tn antigen levels in patients with digestive cancers. *Oncology*, **48**, 321-326.

NAKASAKI, H., MITOMI, T., NOTO, T., OGOSHI, K., HANAUE, H., HAKAMORI, S. (1989). Mosaicism in the expression of tumour-associated carbohydrate antigen in human colonic and gastric cancers. *Cancer Res.*, **49**, 3662-3669.

NARITA, T., FUNAHASHI, H., SATOH, Y., WATANABE, T., SAKAMOTO, J., TAKAGI, H. (1993). Association of expression of blood group-related carbohydrate antigens with prognosis in breast cancer. *Cancer*, **71**, 3044-3053.

PETO, R., PIKE, M.C., ARMITAGE, P., BRESLOW, N.E., COX, D.R., HOWARD, S.V., MANTEL, N., MCPHERSON, K., PETO, J., SMITH, P.G. (1977). Design and analysis of randomised clinical trials requiring prolonged observation of each patient. 2. Analysis and examples. *Br. J. Cancer*, **35**, 1-39.

SIMON, B., PODOLSKY, D.K., MOLDENHAUER, G., ISSELBACHER, K.J., GATTONI-CELLI, S., BRAND, S.J. (1990). Epithelial glycoprotein is a member of a family of epithelial cell surface antigens homologous to nidogen, a matrix adhesion protein. *Proc. Natl Acad. Sci. USA*, **87**, 2755-2759.

SUTHERLAND, R.M. (1988). Cell and environment interactions in tumour microregions: the multicell spheroid model. *Science*, **240**, 177-184.

TAKADA, A., OHMORI, K., TAKAYASHI, N., TSUYOKA, K., YAGO, K., ZENITA, K., HASEGAWA, A., KANNAGI, R. (1991). Adhesion of human cancer cells to vascular endothelium mediated by a carbohydrate antigen, sialyl-Lewis A. *Biochem. Biophys. Res. Commun.*, **179**, 713-716.

YONEZAWA, S., TACHIKAWA, T., SHIN, S., SATO, E. (1992). Sialosyl-Tn antigen. Its distribution in normal adult human tissues and expression in adenocarcinomas. *Am. J. Clin. Pathol.*, **98**, 167-174.